# Association between Herpes Zoster and Osteoporosis: A Nested Case-Control Study Using a National Sample Cohort

**DOI:** 10.1155/2019/4789679

**Published:** 2019-07-30

**Authors:** Chanyang Min, Woo Jin Bang, Dong Jun Oh, Songyong Sim, Hyo Geun Choi

**Affiliations:** ^1^Hallym Data Science Laboratory, Hallym University College of Medicine, Anyang 14068, Republic of Korea; ^2^Department of Otorhinolaryngology-Head & Neck Surgery, Hallym University College of Medicine, Anyang 14068, Republic of Korea; ^3^Graduate School of Public Health, Seoul National University, Seoul 08826, Republic of Korea; ^4^Department of Urology, Hallym University Sacred Heart Hospital, Anyang 14068, Republic of Korea; ^5^Department of Internal Medicine, Asan Medical Center, Ulsan University College of Medicine, Seoul 05505, Republic of Korea; ^6^Department of Statistics & Institute of Statistics, Hallym University, Chuncheon 24252, Republic of Korea

## Abstract

**Objectives:**

Chronic inflammatory disease might affect osteoporosis; however, few studies have reported the association between herpes zoster and osteoporosis. The goal of this study was to estimate the association between herpes zoster and osteoporosis in Korean residents.

**Methods:**

The Korean National Health Insurance Service-National Sample Cohort, which includes individuals aged ≥ 50 years, was assessed from 2002 to 2013. In total, 68,492 osteoporosis participants were matched with 68,492 control participants at a ratio of 1:1 by age, sex, income, and region of residence. We assayed the prior histories of herpes zoster in the osteoporosis and control groups. The diagnoses of herpes zoster and osteoporosis were based on ICD-10 codes and claim codes. Crude and adjusted models of odds ratios (ORs) were explored using conditional logistic regression analyses, and the 95% confidence intervals (CIs) were computed. The participants were stratified according to age, sex, income, and region of residence. Subgroup analyses were performed to investigate the role of age and sex.

**Results:**

The rate of herpes zoster in the osteoporosis group (5.1% [3,487/68,492]) was higher than that in the control group (4.0% [2,738/68,492]). The adjusted OR of herpes zoster in the osteoporosis group was 1.17 (95% CI = 1.11-1.24). In the subgroup analyses, the adjusted OR was 1.34 (95% CI = 1.01-1.78) among males aged < 65 years, 1.20 (95% CI = 1.12-1.29) among females aged < 65 years, and 1.19 (95% CI = 1.04-1.36) among males aged ≥ 65 years.

**Conclusion:**

The ORs of herpes zoster were increased among the osteoporosis patients. This correlation was reliable in all subgroups by age and sex except group of women ≥ 65 years old.

## 1. Introduction

Herpes zoster occurs by recrudescence of the varicella zoster virus, which continues to be latent in neurons in the autonomic ganglia, dorsal root ganglia, and cranial nerve ganglia [1]. After recrudescence, herpes zoster reaches the affected ganglion and peripheral sensory nerve, causing a cellular immunologic reaction, neuronal inflammation, and damage [2]. The incidence of herpes zoster is reportedly 4.0-4.5/1,000 person-years among the entire population [3] and 10-14/1,000 person-years among those aged ≥ 65 years [4–6]. Furthermore, the incidence of herpes zoster is reportedly 10.4/1,000 person-years in Korea [1].

Osteoporosis is a metabolic disorder that involves a destabilized bone turnover rate with high bone resorption compared with bone formation [7]. In the United States, the prevalence of osteoporosis in individuals aged ≥ 50 years is estimated to be 16.0% in men and 29.9% in women [8]. In Korea, the prevalence of osteoporosis in men and women aged ≥ 50 years is approximately 7.5% and 37.3%, respectively [9]. Various issues associated with the turnover of bone structure, including the vitamin D level, bone mass, parathormone, and other metabolites of bone, may be interrupted in osteoporosis patients.

Chronic inflammatory diseases have been previously reported to be the cause of osteoporosis [10], including chronic obstructive pulmonary disease, inflammatory bowel disease, rheumatoid arthritis, systemic lupus erythematosus, and cardiovascular disease [11–15]. Some studies involving a small number of participants have revealed an association between herpes zoster and osteoporosis [16–18]. Only one study reported such an association in a large number of participants from a Taiwanese cohort [19]. The goal of this study was to calculate the correlation between herpes zoster and osteoporosis using a national sample cohort of Korean residents. In the present nested case-control study, we included osteoporosis patients and the same number of control participants who were matched based on potential confounders, such as age, sex, income, and region of residence. We tracked the prior history of herpes zoster in the osteoporosis and control groups.

## 2. Materials and Methods

### 2.1. Study Population and Data Collection

The ethics committee of Hallym University (2017-I102) approved the use of these data. Written informed consent was exempted by the Institutional Review Board. The national cohort data from the Korean Health Insurance Review and Assessment Service-National Sample Cohort (HIRA-NSC) were used in this study.

Enrollment in the Korean National Health Insurence Service (NHIS) is mandatory for all Koreans. Therefore, The NHIS collects the total population database (50 million). From the database, samples of the cohort are selected around 2% using randomized stratified systematic sampling methods to prevent nonsampling errors. The data stratified by 1,476 levels (age [18 groups], sex [2 groups], and income level [41 groups]). This cohort database included (i) personal information, (ii) health insurance claim codes (procedures and prescriptions), (iii) International Classification of Disease-10 (ICD-10) diagnostic codes, (iv) death records from the Korean National Statistical Office (using the Korean Standard Classification of Disease), (v) socioeconomic data (residence and income), and (vi) medical examination data for each participant over a period ranging from 2002 to 2013 (12 years). In addition, all medical treatments in Korea should be followed using the Health Insurance Review & Assessment (HIRA) system without exception. Korean citizens are legally demanded to notice of death to an administrative entity. Causes and the date of death are verified by medical physicians on a death record. The specified reports of these data were described in our previous studies [20, 21].

### 2.2. Participant Selection

Of 1,125,691 cases with 114,369,638 medical claim codes, we selected participants who were diagnosed with osteoporosis. Osteoporosis was diagnosed based on the ICD-10 codes M80, M81, and M82 by a bone density test using X-ray or CT as described in our previous study [22].

Herpes zoster was diagnosed as ICD-10 code B02. Among the identified patients, we only included participants with an ICD-10 code of B02 who visited a hospital or clinic ≥ 2 times or visited a hospital or clinic ≥ 1 time and treated with antiviral medication (n = 64,152).

**Figure 1:**
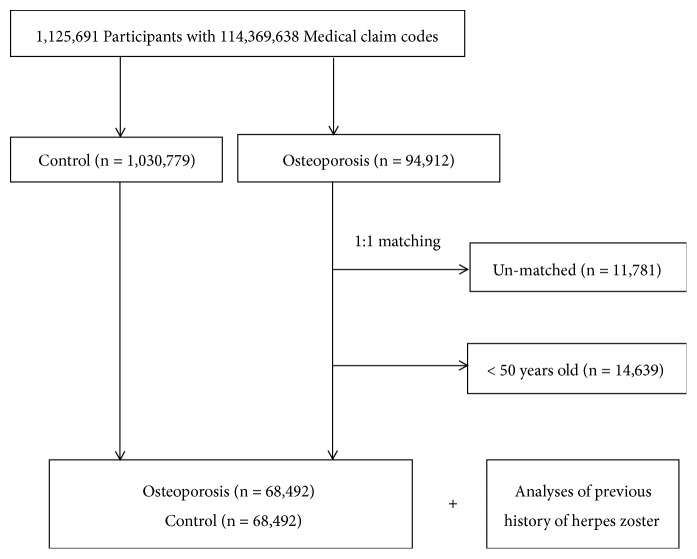
Schematic illustration of the participant selection process that was used in the present study. Of a total of 1,125,691 participants, 68,492 osteoporosis participants were matched with 68,492 control participants by age, sex, income, and region of residence.

The osteoporosis participants were matched at a 1:1 ratio with patients (control group) in this cohort who had never been diagnosed with osteoporosis from 2002 to 2013. The control group included participants from the original population (n = 1,030,779). These subjects were matched by age, sex, income, and region of residence. To avoid selection bias in choosing the matched participants, the control group participants were selected using the method of allocating random numbers and sorted from top to bottom. The matched control participants were presumed to be enrolled at the same time as each matched osteoporosis participant (index date). Therefore, the subjects in the control group who died before the index date were replaced by other control participants. The osteoporosis participants were excluded if they did not have matching control participants (n = 11,781). We also excluded participants aged younger than 50 years (n = 14,639). Our study included participants aged ≥ 50 years because both herpes zoster and osteoporosis are more common after the age of 50 years [22, 23]. Eventually, the 1:1 matching procedure resulted in the inclusion of 68,492 osteoporosis participants and 68,492 control participants (Figure 1).

### 2.3. Variables

The variables of age, sex, income, and region of residence were determined as described in our previous study [24]. The age groups were classified as 50-54, 55-59, 60-64… and 85+ years. The medial use of any steroid (oral or intravenous use) was adjusted as the continuous variable by the sum of using dates.

The Charlson comorbidity index (CCI) was used to identify 17 comorbidities as a continuous variable (0 [no comorbidity] to 29 [multiple comorbidities]) [25].

### 2.4. Statistical Analyses

Chi-square tests and paired* t*-test were used to compare the general features of the participants in the osteoporosis and control groups.

To explore the odds ratio (OR) of herpes zoster with osteoporosis, a conditional logistic regression analysis was used. Crude (simple) and adjusted for CCI and steroids used models were analyzed, and the 95% confidence intervals (CIs) were calculated. The participants were stratified by age, sex, income, and region of residence.

For the subgroup analyses, we categorized the participants by age and sex (age < 65 years and ≥ 65 years; males and females). The clinical characteristics associated with the diseases were analyzed to identify differences based on age and sex.

Two-tailed analyses were performed, and P-values < 0.05 were regarded as indicating significance. The results were assessed using SPSS v. 22.0 (IBM, Armonk, NY, USA).

## 3. Results

The rate of herpes zoster in the osteoporosis group (5.1% [3,487/68,492]) was higher than that in the control group (4.0% [2,738/68,492], P < 0.001, Table 1). The general features (age, sex, income, and region of residence) of the participants were exactly the same due to the matching procedures (P = 1.000). The rates of CCI differed between the osteoporosis and control groups (P < 0.001). The total days of steroids used in osteoporosis group were higher than in control group (P < 0.001).

The adjusted OR of herpes zoster in the osteoporosis group was 1.17 (95% CI = 1.11-1.24) (P < 0.001, Table 2).

In the subgroup analyses, all crude and adjusted ORs of herpes zoster were higher in the osteoporosis group (each P < 0.05) except women aged ≥ 65 years (Table 3). The adjusted OR was 1.34 (95% CI = 1.01-1.78) among males aged < 65 years and 1.20 (95% CI = 1.12-1.29) among females aged < 65 years, 1.19 (95% CI = 1.04-1.36) among males aged ≥ 65 years.

## 4. Discussion

The present study demonstrated that the ORs of herpes zoster in the osteoporosis group were higher than those in the control group (adjusted ORs = 1.17, 95% CI = 1.11-1.24). We found comparable results in the diverse age and sex groups in the stratified analysis. The ORs were lower than those reported in a preceding study using a Taiwanese cohort (hazard ratio, HR = 4.55, 95% CI = 3.09-6.72) [19]. We believe that these differences may be due to the study design and included participants. We excluded participants who were younger than 50 years at the time of the diagnosis of osteoporosis. However, a previous study included a herpes zoster group and a control group comprising 20- to 49-year-old participants who were followed up for 14 years [19]. In that study, the two groups were matched by only age and sex. In our study, we matched not only by age and sex but also by income and region of residence. Matching by these additional factors might have decreased the differences between the patient and control groups.

Previously the association between herpes zoster and osteoporosis is not fully understood pathologically, while some plausible explanation may be possible. Herpes zoster could act as chronic inflammation because it lasts up to 4 weeks [26]. During this period, the cellular immune response provoked by zoster virus results in neuronal inflammation and destruction [27] and could induce vasculopathy [28], such as ischemic infarction or cerebral hemorrhage. This inflammation could cause anorexia, weight loss, fatigue, and depression if it persists as postherpetic neuralgia [27]. Similar to other diseases, this inflammatory process may cause osteoporosis. Previous studies have described that chronic obstructive pulmonary disease, systemic lupus erythematosus, rheumatoid arthritis, inflammatory bowel disease, and cardiovascular diseases can result in secondary osteoporosis [11–15]. Generally, chronic inflammatory diseases affect bone metabolism in three ways [10]. First, inflammatory cytokines elicited by chronic inflammatory diseases can alter bone formation. Interleukin- (IL-) 6 increases osteoclast activity [29], and IL-17 induces proosteoclastogenic cytokines, such as IL-6, tumor necrosis factor- (TNF-) *α*, and receptor activator of nuclear factor kappa-Β ligand (RANKL) [30]. Second, hormonal changes, including changes in leptin and adiponectin, can affect bone loss. High leptin levels can induce apoptosis in human bone marrow stromal cells via the ERK/cPLA2/cytochrome c pathway [31], and adiponectin can increase the fracture risk [32] by stimulating RANKL and inhibiting osteoprotegerin expression in human osteoblasts [33]. Third, malnutrition caused by inflammation can affect osteoporosis. Vitamin D and magnesium deficiencies can also result in osteoporosis [34, 35].

Additionally, the risk of osteoporosis could be accelerated by herpes zoster through vasculitis induced ischemia. Tabrizi et al. demonstrated that herpes zoster infection could induce osteomyelitis [36]. One of the possible pathogeneses is the presence of ischemia through vasculitis induced by herpes zoster [37, 38]. Based on this suggestion, herpes zoster might affect bone health and lead to osteoporosis.

The strength of this study is the utilization of a large, representative, nationwide population sample, which is consistent with our previous studies [39–41]. This study included participants who represent the entire Korean population. The participants were followed up without missing data. Due to the large number of participants, we could randomly select a control group using 1:1 matching by age, sex, income, and region of residence. Additionally, we used an adjusted regression model to reduce confounding effects. This large study could maintain statistical power in the subgroup analysis. Furthermore, this study provides insight regarding the need for the preventive management of osteoporosis in patients with herpes zoster aged ≥ 50 years.

Several limitations should be considered while interpreting our findings. We did not assess the severity of herpes zoster and osteoporosis among the participants. Because herpes zoster and osteoporosis patients do not always visit a clinic, these patients may have been missed. Some patients with mild symptoms may have also been ignored, which could exaggerate our findings. In addition, we could not include all plausible confounding factors between herpes zoster and osteoporosis, such as body mass index, tobacco smoking, menopause status, and dietary habits. Thus, the study outcome should be carefully interpreted because menopause is a risk factor for osteoporosis [42]. Finally, our study could not identify the common pathophysiological mechanism of the association between herpes zoster and osteoporosis because we only estimated the ORs.

## 5. Conclusions

We found increased ORs of herpes zoster in osteoporosis patients compared to the control participants. These associations were reliable in the subgroup analyses by age and sex except group of women ≥ 65 years old.

## Figures and Tables

**Table 1:** General characteristics of the participants.

Characteristics	All participants		
	Osteoporosis	Control group	P-value
Age (years, n, %)			1.000
50-54	12,797 (18.7)	12,797 (18.7)	
55-59	12,491 (18.2)	12,491 (18.2)	
60-64	12,531 (18.3)	12,531 (18.3)	
65-69	11,070 (16.2)	11,070 (16.2)	
70-74	8,496 (12.4)	8,496 (12.4)	
75-79	5,845 (8.5)	5,845 (8.5)	
80-84	3,290 (4.8)	3,290 (4.8)	
85+	1,972 (2.9)	1,972 (2.9)	
Sex (n, %)			1.000
Male	9,740 (14.2)	9,740 (14.2)	
Female	58,752 (85.8)	58,752 (85.8)	
Income (n, %)			1.000
1 (lowest)	13,565 (19.8)	13,565 (19.8)	
2	9,175 (13.4)	9,175 (13.4)	
3	10,730 (15.7)	10,730 (15.7)	
4	14,309 (20.9)	14,309 (20.9)	
5 (highest)	20,713 (30.2)	20,713 (30.2)	
Region of residence (n, %)			1.000
Urban	30,087 (43.9)	30,087 (43.9)	
Rural	38,405 (56.1)	38,405 (56.1)	
CCI (score, n, %)			<0.001*∗*
0	15,450 (22.6)	24,544 (35.8)	
1	3,500 (5.1)	4,397 (6.4)	
2	6,880 (10.0)	6,951 (10.2)	
3	8,986 (13.1)	8,021 (11.7)	
4	9,294 (13.6)	7,449 (10.9)	
5	7,969 (11.6)	5,876 (8.6)	
≥ 6	16,413 (24.0)	11,254 (16.4)	
Total days of steroids used (mean, SD)	107.8 (330.9)	57.9 (176.3)	<0.001†
Herpes zoster (n, %)	3,487 (5.1)	2,738 (4.0)	<0.001*∗*

Abbreviations: CCI, Charlson Comorbidity Index

*∗*Chi-square test. Significance at P < 0.05

†Paired *t*-test. Significance at P < 0.05

**Table 2:** Crude and adjusted odd ratios (95% confidence interval) of osteoporosis and herpes zoster.

Characteristics	Herpes zoster			
	Crude†	P-value	Adjusted†‡	P-value
Osteoporosis	1.29 (1.23-1.36)	<0.001*∗*	1.17 (1.11-1.24)	<0.001*∗*
Control	1.00		1.00	

*∗* Conditional logistic regression analyses. Significance at P < 0.05

† Stratified based on age, sex, income, and region of residence.

‡ Adjusted model for the Charlson Comorbidity Index score and steroids used.

**Table 3:** Subgroup analysis of crude and adjusted odd ratios (95% confidence interval) of osteoporosis and herpes zoster according to age and sex.

Characteristics	Herpes zoster			
	Crude†	P-value	Adjusted†‡	P-value
Age < 65 years, men (n = 5,620)				
Osteoporosis	1.39 (1.06-1.81)	0.016*∗*	1.34 (1.01-1.78)	0.043*∗*
Control	1.00		1.00	
Age < 65 years, women (n = 70,018)				
Osteoporosis	1.29 (1.20-1.39)	<0.001*∗*	1.20 (1.12-1.29)	<0.001*∗*
Control	1.00		1.00	
Age ≥ 65 years, men (n = 13,860)				
Osteoporosis	1.34 (1.18-1.52)	<0.001*∗*	1.19 (1.04-1.36)	0.011*∗*
Control	1.00		1.00	
Age ≥ 65 years, women (n = 47,486)				
Osteoporosis	1.25 (1.14-1.38)	<0.001*∗*	1.09 (0.99-1.21)	0.093
Control	1.00		1.00	

*∗* Conditional logistic regression analyses. Significance at P < 0.05

† Stratified based on age, income, and region of residence.

‡ Adjusted model for the Charlson Comorbidity Index score and steroids used.

## Data Availability

The current article used a national sample cohort and does not involve data that can be available.
